# Supplementary material for: A simple technique to improve performance of four-port wideband MIMO antenna using shorting pins
Source: PLoS One. 2025 May 23;20(5):e0321602. doi: 10.1371/journal.pone.0321602 (PMC12101636; doi:10.1371/journal.pone.0321602)
Supplement: Attachment — Submitted filename: Detailed Comments_PONE-D-24-38256.pdf [file pone.0321602.s001.pdf]

A. Novelty and Unique Contributions:

The manuscript addresses a well-known issue in MIMO antenna design, which is mutual coupling between closely placed antenna elements. The proposed solution of using shorting pins to improve isolation between elements, while maintaining a compact design, brings a modest level of novelty. Specifically:

1. **Use of Shorting Pins**: Shorting pins as a decoupling mechanism have been used before, but their implementation inside the MIMO elements without requiring additional space is a unique contribution.

2. **Combination of Slot and Metasurface (MS) for Wideband Operation**: While metasurfaces have been employed for bandwidth enhancement, the integration of slot and MS resonances in a low-profile, compact design is innovative.

3. **Compact Design with High Isolation**: The design achieves good isolation and bandwidth while maintaining a small footprint ($1.08\lambda \times 0.89\lambda \times 0.07\lambda$). This compactness, especially with a center-to-center element spacing of 0.35λ, is another strong point of the work.

However, the novelty is incremental rather than groundbreaking. Many of the techniques discussed, such as orthogonal polarization and metasurfaces for bandwidth enhancement, are well-established in the literature.

B. Content Review:

1. **Abstract**:
    - The abstract succinctly summarizes the proposed design and its key performance metrics. It mentions the compact size, wideband operation, and isolation, but it could benefit from a clearer statement of novelty to make the unique aspects stand out more explicitly.

2. **Introduction**:
    - The introduction provides a good overview of the problem (mutual coupling and wideband operation) and discusses previous solutions. It is well-structured, but the novelty of the proposed work could be more clearly emphasized.
    - Citations cover a wide range of related work, but it would be beneficial to mention more recent approaches to compact MIMO designs, especially in terms of practical applications for 5G and beyond.

3. **Technical Soundness**:
   - **Design Methodology**: The methodology of integrating slots and metasurfaces is sound, and the simulations support the claimed improvements. However, the design choices, such as the specific placement of shorting pins, could benefit from a deeper theoretical justification beyond the observed simulation results.
   - **Figures and Diagrams**: Figures (e.g., Fig. 1, 2, 5, 6) provide clear visual representations of the design and performance. The geometry and current distribution figures are particularly useful for understanding the design's effectiveness.
     - The radiation patterns (Fig. 10 and Fig. 18) show that while broadside radiation is achieved, there is some beam tilt. This could be discussed more thoroughly, especially regarding the practical implications for real-world usage.
4. **Results**:
   - **Bandwidth and Isolation**: The achieved bandwidth of 4.9 to 5.8 GHz (16.8%) with isolation above 20 dB is strong. However, the bandwidth is somewhat narrow compared to the latest wideband MIMO antennas. Is there any potential to further enhance this? Discussing this limitation in more detail would add depth.
   - **Gain**: The maximum broadside gain of 4.5 dBi is reasonable for the proposed size, but it is not particularly high. The trade-off between gain and size could be more explicitly discussed.
   - **Measurement vs. Simulation**: The manuscript acknowledges slight discrepancies between simulated and measured results (Fig. 16 and 17), which is typical in such designs. However, further explanation of the causes (e.g., substrate tolerance, fabrication imperfections) would strengthen the argument.
5. **Novel Contributions Section**:
   - A dedicated section or clearer presentation of the unique aspects of the design would help the reader quickly grasp the novel elements, especially in comparison with existing solutions. This is partially covered in the performance comparison table (Table 1), but more emphasis on the compactness, isolation, and slot/MS combination would add clarity.
6. **Tables**:
   - **Table 1**: The performance comparison table is well-constructed, showing that the proposed antenna has competitive characteristics in terms of size, bandwidth, and isolation. It demonstrates that despite its compact size, the proposed design achieves similar or better performance compared to larger antennas.

C. Strengths:

- **Compact Design**: The small footprint and low profile make this antenna suitable for space-constrained applications, such as mobile devices or compact IoT systems.
- **High Isolation**: The shorting pin technique effectively reduces mutual coupling, as shown by the isolation improvement in the S-parameters.
- **Simulation Support**: The manuscript is well-supported by simulation and measurement results, which align closely in most cases.

D. Areas for Improvement:

1. **Theoretical Analysis**: The manuscript would benefit from a deeper theoretical discussion of how the shorting pins and slot/MS combination work together to enhance bandwidth and isolation.
2. **Practical Applications**: While the manuscript is technically sound, it could be strengthened by discussing practical implications, such as real-world deployment in 5G or Wi-Fi 6E systems. How would this design perform under different environmental conditions or in a densely populated MIMO system?
3. **Beam Steering**: The tilt of the radiation pattern is noted but not deeply analyzed. Could this be mitigated with further design modifications?

E. Conclusion:

The manuscript presents a solid, incremental contribution to MIMO antenna design, particularly in terms of compactness, isolation, and bandwidth enhancement using shorting pins and a slot/MS combination. While the novelty is modest, it provides practical improvements for modern communication systems. Some additional theoretical depth and discussion of practical applications would enhance the impact of the work.
